# The Ethylene Biosynthetic Enzymes, 1-Aminocyclopropane-1-Carboxylate (ACC) Synthase (ACS) and ACC Oxidase (ACO): The Less Explored Players in Abiotic Stress Tolerance

**DOI:** 10.3390/biom14010090

**Published:** 2024-01-11

**Authors:** Sheen Khan, Ameena Fatima Alvi, Sadaf Saify, Noushina Iqbal, Nafees A. Khan

**Affiliations:** 1 Plant Physiology and Biochemistry Laboratory, Department of Botany, Aligarh Muslim University, Aligarh 202002, India; khansheen192@gmail.com (S.K.); saifysadaf94@gmail.com (S.S.); 2 Department of Botany, Jamia Hamdard, New Delhi 110062, India; naushina.iqbal@gmail.com

**Keywords:** ethylene biosynthesis, ACS and ACO regulation, abiotic stress, nutrient starvation, growth and development

## Abstract

Ethylene is an essential plant hormone, critical in various physiological processes. These processes include seed germination, leaf senescence, fruit ripening, and the plant’s response to environmental stressors. Ethylene biosynthesis is tightly regulated by two key enzymes, namely 1-aminocyclopropane-1-carboxylate synthase (ACS) and 1-aminocyclopropane-1-carboxylate oxidase (ACO). Initially, the prevailing hypothesis suggested that ACS is the limiting factor in the ethylene biosynthesis pathway. Nevertheless, accumulating evidence from various studies has demonstrated that ACO, under specific circumstances, acts as the rate-limiting enzyme in ethylene production. Under normal developmental processes, ACS and ACO collaborate to maintain balanced ethylene production, ensuring proper plant growth and physiology. However, under abiotic stress conditions, such as drought, salinity, extreme temperatures, or pathogen attack, the regulation of ethylene biosynthesis becomes critical for plants’ survival. This review highlights the structural characteristics and examines the transcriptional, post-transcriptional, and post-translational regulation of ACS and ACO and their role under abiotic stress conditions. Reviews on the role of ethylene signaling in abiotic stress adaptation are available. However, a review delineating the role of ACS and ACO in abiotic stress acclimation is unavailable. Exploring how particular ACS and ACO isoforms contribute to a specific plant’s response to various abiotic stresses and understanding how they are regulated can guide the development of focused strategies. These strategies aim to enhance a plant’s ability to cope with environmental challenges more effectively.

## 1. Introduction

**Figure 1:**
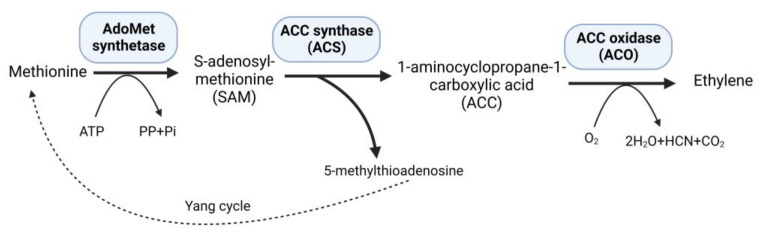
Ethylene biosynthesis pathway.

Ethylene, a versatile phytohormone known for its multifaceted regulatory functions in plant growth and development, also influences critical processes, such as seed germination, root growth, fruit ripening, and flower and leaf abscission. The research on ethylene responses is documented in a large number of studies [1]. The early research on ethylene shows that its effects on plants dates back to the 1800s. The first case of illuminating gas affecting plants was shown in 1858 [2]. A review published in 2015 summarized the history of ethylene research, including biosynthesis, regulation, signaling, and physiological effects on plants [3]. Moreover, the research on ethylene advanced with the passage of time, and researchers participated to unravel the role of ethylene in plants under stressful environments. Plants subjected to persistent stressful environments in their natural habitat exhibit stress avoidance strategies and establish mechanisms to withstand and endure stress, thereby developing stress tolerance. Studies on higher plants suggest increased levels of ethylene production in response to abiotic and biotic stresses [4]. At low concentrations, ethylene can promote plant growth and development; however, when ethylene levels rise, as frequently observed under stressful situations, it may have negative consequences with aberrant plant growth and development [5]. Under stress conditions, elevated levels of 1-aminocyclopropane-1-carboxylate (ACC) synthase (ACS) stimulate the production of increased amounts of the substrate 1-aminocyclopropane-1-carboxylate (ACC), consequently leading to higher ethylene synthesis within plant tissues [6]. Methionine is converted to S-adenosyl-L-methionine (SAM) by the enzyme SAM synthetase, which is part of plants’ well-established ethylene production route. Subsequently, ACS catalyzes the conversion of SAM to ACC, generating 5-methylthioadenosine (MTA) as a byproduct, which is then recycled back into methionine through a multi-step process known as the Yang cycle, while ACC oxidase (ACO) converts ACC into ethylene [7] (Figure 1).

Early research suggested that ACS serves as the rate-limiting enzyme, which prompted a substantial investigation into the control of ACS protein activity and stability [8]. However, a rising body of evidence has accumulated, indicating that ACO is the limiting factor in ethylene synthesis during specifically dedicated processes [9]. This conveys that ACS and ACO are important for ethylene biosynthesis and plant function regulation. ACS is an intracellular protein located in the cytosol and distinguished by its short lifespan and dependence on the cofactor pyridoxal-5′-phosphate (PLP) for enzymatic activity [8]. Wang et al. [10] reported the regulation of ethylene biosynthesis through WRKY29, which transactivates the expression of ACS and ACO and brings about a pleiotropic effect on plant growth and development. ACS is a multiple-gene-encoding polypeptide that varies from species to species. For instance, eight *ACS* genes in *Lycopersicum esculentum* [11] and five in *Oryza sativa* and *Solanum tuberosum* are reported [12]. Environmental factors differentially regulate the expression of each *ACS* throughout the plant life cycle. However, there are 12 *ACSs* reported in the Arabidopsis genome, out of which only 8 (*ACS 2*, *4–9*, *11*) are enzymatically active. These genes have shown tolerance responses in plants under various abiotic stress. *AtACS7*, *ACS9*, and *ACS11* maintain a balanced relationship between ethylene, ROS, and brassinosteroid phytohormones [13]. Additionally, *AtACS2* and *AtACS5* participate in pathways that respond to abscisic acid (ABA) and control plant growth and development [14]. Under hypoxia, the tissue-specific expression response of *OsACS1* and *OsACS3* is reported in etiolated seedlings in shoots and roots, respectively, while *OsACS2* is mainly expressed in roots and downregulated by hypoxic conditions. During submergence, OsACS5 mRNA is found to accumulate in the vascular bundle of young stems and leaf sheaths [15]. The phosphorylation of serine residues at sites 476 and 479 in the C-terminal region of *MaACS1* is an essential regulatory mechanism for *Musa paradisiaca* fruit maturation [16]. Previous research has indicated that the upregulation of *ACS* genes increases the synthesis of defensive proteins, paving the way for ACC production followed by ethylene [17]. 

Similarly, as mentioned earlier, ACO is subject to strict regulation. The subcellular localization of ACO is contentious, with conflicting studies proposing either plasma membrane or cytosolic localization. It exhibits diverse expression levels in both vegetative and reproductive tissues, playing a crucial role in limiting the rate of ethylene biosynthesis [9] Evidence showed the role of ACO in abiotic stress tolerance; for instance, flooding induces the upregulation of *StACO1* and *StACO2* in potatoes, with *StACO1* exhibiting high sensitivity to this stress [18]. In deep water rice, *OsACO1* plays a role in internode elongation, and submergence enhances both ACO activity and *OsACO1* mRNA levels [19,20]. A study on tomatoes revealed that ethylene-induced hydrogen sulfide production through persulfidation of LeACO1 and LeACO2 reduces the activity of enzyme and ethylene production, thus helping in osmotic stress tolerance [21]. Moreover, *ACO* in Arabidopsis shows tissue-specific expression patterns, meaning its differential expression is required for optimum ethylene production at different phases of the plant life cycle [9]. Though ethylene has been reported as a major phytohormone influencing plant growth potential under abiotic stress, it is equally relevant to highlight the role of ACS and ACO expression under various abiotic stresses to know the root cause of abiotic stress responses. This review is an effort to highlight the more profound role of ACS and ACO in intimating plant physiology and development under abiotic stress conditions.

## 2. Structure and Regulation of ACS

### 2.1. ACS

The ACS protein is located in the cytosol. It is an enzyme that depends onPLP and is evolutionarily related to the aminotransferase superfamily. It also requires pyridoxal as a cofactor [22]. The structure of ACS involves two domains. The αβα sandwich domain consists of a core seven-strand β-sheet surrounded by nine α-helices (residues 82–322 or H3–H11). On the other hand, αβ domaincomprises five α-helices and β-strands (residues 11–19, 25–77, and 323–438). The dimer form of ACS is considered the basic catalytic unit, with the C-terminal helix H14 contacting the N-terminal residues 11–19. The PLP cofactor is anticipated and located between the two domains. However, it was also observed that a monomeric ACS may also be catalytically active [23].

Most PLP-dependent enzymes generally have a lysine residue in their active sites. The presence of Lys278 in the tomato ACS is conserved among other ACS isoforms, and this particular residue plays a crucial role in binding PLP by facilitating a reduction in the double bond between PLP and the enzyme. Through crystal structure analysis, it was determined that the active site of ACS comprises crucial amino acid residues, including Tyr85, Thr121, Asn202, Asp230, Tyr233, Ser270, Lys273, Arg281, and Arg407. These amino acids serve as the enzyme’s homodimer interface and are crucial for PLP binding [24]. The aminotransferase subfamily called ACS-like encompasses two categories: true ACS isozymes and related proteins that do not possess ACS enzymatic activity [25]. Out of 12 *ACS* genes in Arabidopsis, *ACS 3* is a pseudogene, whereas *ACS10* and *12* are Asp, Phe, and Tyr aminotransferases, not ACSs, forming a putative aminotransferase clade [26]. Enzymatically active ACSs (*ACS 2*, *4–9*, *11*) are divided into three types based on the presence or absence of a phosphorylation site at the C-terminal [25].

### 2.2. Transcriptional and Post-Transcriptional Regulation of ACS

The regulation of ethylene production involves the differential transcription of ACS genes in response to developmental changes and various external stimuli [27]. The tomato ACS gene family, for instance, consists of at least eight genes controlled by various biotic and abiotic stimuli. Two systems of ethylene production are present in tomatoes and other climacteric plants: system 1 is autoinhibitory, inhibiting ethylene biosynthesis during vegetative development, and system 2 is autocatalytic, driving ethylene production during fruit ripening and petal senescence. Once initiated, this autocatalytic system forms a positive feedback loop that integrates fruit ripening. The differential expressions of four *ACS* genes in tomatoes are required to transition from system I to system II during fruit maturation [11]. *LeACS1A* and *LeACS6* have been suggested to play a role in ethylene production in system 1 in unripe fruits and vegetative tissues, exhibiting ethylene at baseline levels. At the same time, *LeACS1A* expression increases, and *LeACS4* is induced when the fruit becomes ripening competent. On the other hand, system 2’s ethylene biosynthesis is initiated and sustained through the ethylene-responsive expression of *LeACS2* and *LeACS4* during fruit ripening and floral senescence [28]. Recently, the transcription factor MdERF4, activated by H_2_O_2_, exerted negative control on ethylene production during fruit ripening by inhibiting the transcription of *MdACS1* [29]. In apples, MdAGL30, MdAGL104, MdERF008, MdNAC71, MdDof1.2, MdHSFB2a, and MdHSFB3 were found to interact with the promoter of *MdACS1* and directly control its transcription, thus regulating ethylene production [30]. The MADS-box transcription factor SlRIN was the first identified regulator of *ACS* expression, specifically enhancing the expression of certain tomato ACS genes [31]. Furthermore, there is supporting evidence demonstrating the direct control of *ACS* transcription by ethylene. For instance, the interaction of the tomato ethylene response factor SlERF2/TERF2 with the *NtACS3* promoter induced its expression when expressed in *Nicotiana tabacum* [32]. In *Poncirus trifoliata*, PtrERF9 plays a role in regulating ethylene production by activating the *PtrACS1*. This creates a feedback loop that strengthens the transcriptional control of its target genes, potentially enhancing cold tolerance [33]. Also, the Arabidopsis WRKY29 transcription factor enhances the expression of *ACS5/6/8/11* and *ACO5*, promoting basal ethylene production in roots [10]. In kiwifruit transcription factors, AcNACs (1–4) upregulate *AcACS1*, while AdERF105L and AdWRKY29 downregulate *AdACS1/2*, thus regulating ethylene biosynthesis in wounding and fruit ripening [34,35]. It is widely acknowledged that auxins, cytokinins, brassinosteroids, and abscisic acid can potentially impact ethylene production [6]. Most *ACSs* show transcriptional induction in response to auxin, with multiple AuxREs (auxin response elements) identified in several auxin-regulated *ACSs* in Arabidopsis and melons. However, the induction pattern of *ACSs* by auxin is complex and varies spatially [36,37,38]. Different *ACSs* exhibit cell-type specificity in their response to auxin, with *ACS8* expands its expression into specific cell layers such as the epidermis and protoxylem, with *ACS11* expanding its expression in the root’s cell division zone [36]. Moreover, most *ACSs* display transcriptional activation in response to cycloheximide, indicating the presence of a transient repressor protein that suppresses *ACS* transcription. The Aux/IAA proteins, known for their short half-life and negative regulation of auxin responses, are potential candidates for such repressors [27]. Interestingly, *ACS1*, the only *ACS* that did not exhibit induction in response to auxin, also failed to respond to cycloheximide treatment [36]. The exploration of transcription factors governing the regulation of ACS under abiotic stress remains incomplete. While Table 1 presents some documented transcription factors influencing *ACSs* in this context, a substantial need exists for further investigation to discern the specific transcription factors that impact individual *ACS* isoforms in response to abiotic stress.

Until recently, there was little knowledge accessible on the role of post-transcriptional mechanisms in controlling ethylene production. Small, non-coding RNA molecules called microRNAs (miRNAs) participate in the post-transcriptional control of plant growth and developmental processes [47]. The overexpression of *miR1917* substantially increased the level of expression of *ACS2* and *ACS4*, resulting in ethylene response phenotypes, including triple response, leaf petiole curvature, pedicel abscission rate, and fruit maturation [48]. It was observed that *tae-miR2275-3p* is involved in early meiosis, and it targets the *TaACS8*, *TaACS11*, and *TaACS12*, meaning that these could play a role in early meiosis in wheat by post-transcriptionally regulating ethylene biosynthesis [49]. Post-transcriptional cleavage of *ACS4* by *miR843* and *ACS4/8* by *miR1591a* helps in regulating ethylene biosynthesis in Arabidopsis [50]. Acetosalicylic acid affects ethylene biosynthesis at the post-transcriptional level by downregulating mitogen-activating protein kinase *AdMPK16*, which phosphorylates and stabilizes *AdACS3* in *Actinidia* [35].

### 2.3. Post-Translational Regulation of ACS

**Figure 2:**
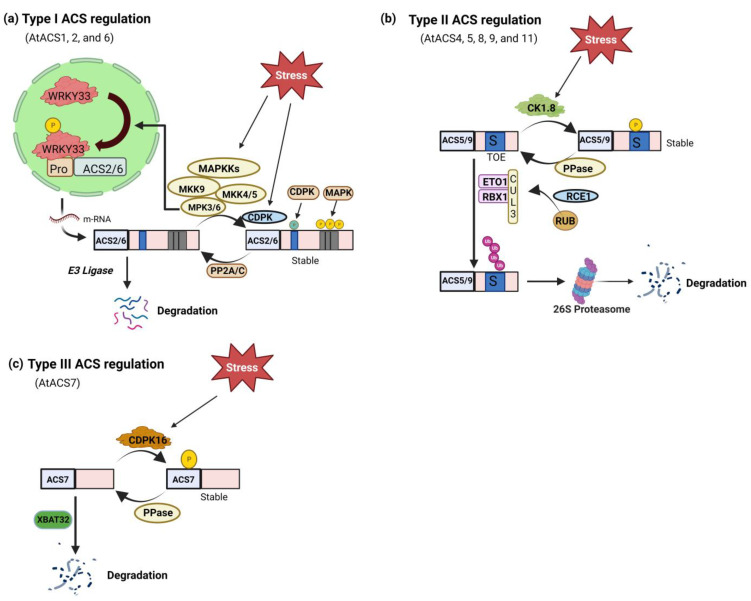
Post-translational regulation of ACS. (**a**) Arabidopsis type I ACS transcriptional and translational control by stimuli-responsive MAPK3/6 (mitogen-activated protein kinase) pathway. The MPK3/MPK6 phosphorylation of ACS2/ACS6 results in the stability of the ACS protein. In addition, MPK3/MPK6 activation through WRKY33, another MPK3/MPK6 substrate, similarly upregulates the expression of the ACS2 and ACS6 genes. Cellular ACS activity and ethylene production are significantly increased by dual-level modulation of Type I ACS by MAPKs and CDPKs (calcium-dependent protein kinase). There have also been discovered phosphatases (PP2A/C) that play a role in the dephosphorylation of ACS2/ACS6. (**b**) ETO1 (ETHYLENE OVERPRODUCER 1) containing E3 ligases that are capable of recognizing the TOE (Target of ETO1) domain in the C-termini of Type II ACSs, such as Arabidopsis ACS5, stabilize these proteins. The stability of Type II ACS protein is hypothesized to be controlled by CDPK phosphorylation, which is thought to play a role in this ubiquitination process. (**c**) Type III ACS isoform phosphorylation and stability modulation. A CDPK may phosphorylate the catalytic domain of the Type III ACS Arabidopsis ACS7, which is thought to be involved in ethylene production during root gravitropism. The ubiquitin-26S proteasome pathway, which requires the XBAT32 (XB3 orthologs 1 in *A. thaliana*) E3 ligase, may degrade ACS7.

In addition to transcriptional regulation, modifying ACS proteins after translation plays a vital role in controlling ethylene production. ACS protein stability and turnover, governed by kinases, phosphatases, and the ubiquitin–proteasome system, are pivotal for ethylene production during development and stress responses [25]. Particular amino acid residues in the ACS proteins are necessary for regulating post-translational modifications. The peptides located in the C-terminal regions of three different subfamilies of ACS proteins facilitate their differentiation: Type 1 ACS proteins contain target sites for CDPK and MAPK (Figure 2a), while Type 2 ACS proteins possess E3 ligases and CDPK (Figure 2b). However, no such sites are found in Type 3 ACS proteins (Figure 2c) [51]. The detailed mechanism of post-translational regulation of ACS is mentioned below.

#### 2.3.1. Regulation of ACS by Phosphorylation and Dephosphorylation

The pivotal regulation of ACS protein stability depends on the dynamic phosphorylation and dephosphorylation processes. In tomato cells, exposure to protein kinase inhibitors impedes elicitor-induced ACS and ethylene biosynthesis stimulation. Conversely, the inhibition of protein phosphatase leads to a rapid increase in ACS activity, even without elicitors. Notably, these modifications do not directly impact the catalytic efficacy of the enzyme but instead modulate ACS activity by influencing its turnover rate [52].

Detailed analysis showed that phosphorylation of ACS2/ACS6 by MAPK3/MAPK6 (mitogen-activated protein kinase) enhances the stability of ACS proteins, resulting in increased cellular ACS activity and elevated ethylene generation [53]. Additionally, the phosphorylation of specific serine residues in the C-termini of ACS2/ACS6, which prevents their degradation by unidentified ubiquitin–proteasome machinery [54]. In response to pathogen infection, MPK3 and MPK6 also control the expression of *ACS2* and *ACS6* by phosphorylating the transcription factor WRKY33. This double-level regulation of ethylene induction affects both the stability of proteins and the expression of genes [55].

Studies suggest that CDPKs (calcium-dependent protein kinases) regulate the turnover of ACS proteins. The veracity of this notion was supported by studies demonstrating that the administration of inhibitors targeting Ca^2+^ channels and calmodulin-binding resulted in a decline in the ethylene-induced expression of *ACO2* and *ACS2* in *Pisum sativum* seedlings [56]. Corresponding findings were observed in *Vigna radiata* seedlings, where the expressions of *VrACS1* and *VrACO1*, along with the activity of *VrACO1*, were diminished following supplementation with inhibitors of Ca^2+^ [57]. Another report demonstrated that in tomatoes, LeACS2 turnover was regulated by phosphorylation by LeCDPK2 [58]. Similarly, *Gossypium hirsutum*, GhACS2, was phosphorylated by GhCDPK1, resulting in increased enzymatic efficiency [59]. Serine residue, Ser-460, at the C-terminal, serves as the target for phosphorylation by CDPK, and this phosphorylation site is conserved in both type I and type II ACS proteins [27]. In Arabidopsis, CDPK4 and CDPK11 were shown to phosphorylate ACS6, leading to the stabilization of the ACS6 protein and enhanced ethylene evolution during root growth [60]. These findings highlight the crucial role of CPK phosphorylation in regulating the stability of type I and II ACS proteins. Despite lacking known regulatory sequences in their short C-termini, Arabidopsis ACS7 (type III) can still be phosphorylated by a CDPK in vitro within its catalytic domain. This phosphorylation involves ethylene production during root gravitropism [61]. ACS7 is also subject to degradation through the Ub-26S proteasome pathway mediated by the XBAT32 E3 ligase [62]. Nevertheless, it is currently unclear whether the phosphorylation of ACS7 by CDPK(s) interacts with or opposes its proteasomal degradation regulation [63].

Studies reveal that regulatory factors target different types of ACS proteins, indicating the rapid evolution of regulatory mechanisms. For example, the CK1.8-mediated destabilizing phosphorylation site (T463) is present in certain ACS types (ACS5, ACS9, and some type 1 isozyme) but absent in others (ACS4, ACS8). Additionally, interaction with 14-3-3 proteins stabilizes at least one isozyme from each ACS type (ACS2, ACS5, ACS7). The precise mechanism of 14-3-3 stabilization for ACS2 and ACS7 remains unknown. In the case of ACS5, 14-3-3 interaction counteracts ETO/EOL-mediated turnover by stabilizing ACS protein, and destabilizing ETO/EOL proteins was not dependent on the TOE sequence of ACS5 [64]. These findings highlight the complexity and versatility of the regulatory network controlling ethylene synthesis.

The protein phosphatase 2A RCN1 (roots curl in 1-N-naphthylphthalamic acid) destabilizes ACS6 (type-I), resulting in a slower turnover rate in the *rcn1* mutant. Conversely, the presence of the rcn1 mutation reduces the stability of ACS5 (type-II), demonstrating that RCN1 and the phosphorylation/dephosphorylation process regulate ACS stability in a type-specific manner [65]. Similarly, ABI1 (ABA-insensitive 1), a protein phosphatase, dephosphorylates MAPK6 and the C-terminus of ACS6 (type-I), destabilizing ACS6. Additionally, ABI1 and ABI1-like protein phosphatase 2C are implicated in directly interacting with ACS7 to regulate ethylene biosynthesis [66]. The overexpression of *AP2C1*, a gene encoding a Ser/Thr protein type 2C phosphatase, can negatively regulate MPK6, suppressing wounding-induced ethylene production [67]. It can be inferred from the above reports that phosphatases target Type I ACSs, MPK6, and potentially MPK3 to counteract the phosphorylation-mediated stabilization of ACS proteins [63] (Figure 2a).

#### 2.3.2. ACS Protein Turnover Ubiquitin–Proteasome Degradation System

Arabidopsis *ethylene-overproducer* (*eto*) mutants demonstrated the involvement of the ubiquitin 26S proteasomal pathway in posttranslational ACS protein regulation. Among the three mutants, dominant mutations, *eto2* with a frameshift mutation in ACS5 C-terminus and *eto3* with a missense mutation in ACS9 C-terminus, stabilize the respective protein [68,69]. The *eto1* mutant had a recessive mutation [69]. The *ETHYLENE OVERPRODUCER1* (*ETO1*) gene was identified as a component involved in the turnover of ACS5 protein. ETO1 encodes an E3 ligase component with a BTB domain (broad-complex/tramtrack/ bric-a-brac) [63]. BTB proteins are substrate-specific adaptors that connect the substrate and the scaffold component, CUL3, in SCF-type ubiquitin E3 ligases by acting as the chimeric modules of SKP1 and F-box proteins [70]. In vitro experiments have provided evidence showing that ETO1 acts as an adaptor protein, directly interacting with ACS5 and CUL3. This interaction enables ETO1 to specifically target ACS5 for degradation. Two mechanisms were proposed to show the negative regulation of ETO1 on ACS5 activity. First, the ETO1 protein interacts with the C-terminus of ACS5 to either mask catalytic sites or modify the protein’s conformation, affecting substrate access. This interaction may also dissociate or destabilize the functional dimer of ACS5 [71]. Biochemical studies and crystal structure analysis suggest that ACS enzymes are a dimer with shared active sites from each monomer [24], suggesting that dimerization may provide optimal enzyme activity and stability. In the second model, ETO1 interacts with CUL3 and facilitates ACS5 protein degradation via the ubiquitin pathway, but whether it interacts with ACS5 monomers or dimers is unclear. ETO1 disruption results in enhanced stability of the ACS5 protein and elevated ethylene biosynthesis [69]. Within Arabidopsis, ETO1 is accompanied by two closely related paralogs, EOL1 and EOL2 (ETO1-LIKE), that collectively reduce ethylene production [72]. These BTB proteins selectively focus on type II ACS proteins, which possess a distinctive C-terminal TOE domain recognized by ETO1/EOL1/EOL2 [72]. Light exposure destabilizes ETO1 and EOLs, stabilizing ACS5 protein and presenting a novel regulatory mechanism for ethylene synthesis in response to environmental stressors [64]. The critical importance of the C-terminal sequence of Type II ACS proteins in relation to their stability becomes evident when considering the findings from both the cloning of ETO1 and *eto2*/*eto3* mutants.

Mutants in the E3 ligase pathway provide additional evidence for the role of E3 ligase components in regulating ACS stability [63]. Research findings indicate that the function of the E3 ligase is regulated by a small peptide known as RUB (related to ubiquitin), which shares sequence similarity with ubiquitin. Reducing the function of RUB1 and RUB2 through RNA interference led to increased ethylene production and a partial triple response in etiolated seedlings [73]. The rapid turnover of ACS proteins requires the conjugation of RUB to CUL3, which modifies the activity of the E3 ligase containing ETO1. This concept was supported by studying the stability of ACS proteins in *dsrub* lines [74]. Another alteration in ethylene biosynthesis was observed in a recessive mutation of *RCE1* (*RUB1-CONJUGATING ENZYME 1*), which encodes the RUB1 conjugating enzyme responsible for attaching RUB1 to the SCF-type E3 ligase complex. Like the *dsrub* lines, the *rce1* mutant exhibited a partial triple response due to excess ethylene [75]. While there was no indication of increased ACS activity in the hypocotyls of *rce1* mutants, there was a slight increment in ACO activity. The authors proposed a scenario in which the enhanced ethylene synthesis in mutants is attributed to enhanced ACO activity rather than increased ACS activity [75] (Figure 2b).

XBAT32 (XB3 ortholog 2 in *A. thaliana*), a RING domain of the type-E3 ligase subfamily, has a sequence of ankyrin repeats that regulate the turnover of ACS4 (type-2) and ACS7 (type-3) [62]. Recently, it was documented that GhXB38D, E3 ubiquitin ligase, acts as a negative regulator pf fiber elongation in cotton by facilitating the ubiquitination of *GhACS4* and *GhACO1* [76]. In an *xbat32-1* mutant, the degradation rate of ACS7 is significantly reduced relative to the wild type, indicating the involvement of XBAT32 in ACS7 turnover. Interestingly, the ubiquitin degradation system may still control the degradation of ACS7, even if its C-terminal lacks known regulatory regions, suggesting the existence of unexplained cis-regulatory sequences [63] (Figure 2c).

A recent study showed that RING E3 ligase MaXB3 (*Musa acuminata* XA21 binding protein 3) regulates the protein turnover of MaACS1 and MaACO1. The interaction between MaXB3 and MaNAC2, a positive modulator of ethylene biosynthesis, facilitates the upregulation of *MaACS1* and *MaACO1* gene expression and the ubiquitination of MaNAC2. Additionally, MaNAC1 and MaNAC2 negatively regulate the expression of *MaXB3*, ensuring the balance of MaNAC2, MaACS1, and MaACO1 protein levels. These discoveries exemplify the regulatory mechanism plants utilize to regulate ethylene biosynthesis, employing a combination of post-translational and transcriptional control [77]. However, no specific E3 ligase targeting Type I ACS proteins has been identified. The application of MG132, a 26S proteasome inhibitor, results in a notable enhancement in ACS6 stability, suggesting the participation of the Ub-proteasome pathway in regulating Type I ACS protein stability [63].

#### 2.3.3. Other Mechanisms for ACS Regulation

Phytohormones play crucial roles in regulating various signals in ethylene production necessary for growth and development through their cooperative or inhibitory interaction with other hormones [78]. The stability of ACS protein is subject to regulation by a range of hormonal regulators, including cytokinins, brassinosteroids, auxins, jasmonic acid, abscisic acid, salicylic acid, and gibberellic acid [79]. For instance, the *cin5* mutant represents the first in a series of *cin* mutants (cytokinin insensitive) of Arabidopsis and is identified as a loss-of-function allele of the *ACS5*. This mutant exhibits significant insensitivity to exogenous cytokinin, leading to a failure in displaying the triple response. Interestingly, the *cin5* mutant still demonstrates a normal triple response to ethylene, indicating that ACS5 is the primary target for cytokinin-mediated ethylene induction in etiolated seedlings [80]. Cytokinin triggers ethylene synthesis by influencing the stability of ACS proteins, thereby enhancing overall ethylene production in plants [80]. Elevated levels of ABA in Arabidopsis promote the upregulation of *ABI4* expression, suppressing ethylene generation by reducing the transcription of *ACS4* and *ACS8*, leading to stomatal closure. This indicates an intricate interplay between the hormones regulating stomatal aperture size [78]. Zhu et al. [81] elucidated this mechanism in tomatoes, showing that applying brassinolide (BL) to tomato plants increases ethylene and H_2_O_2_ production. This effect is attributed to the augmentation of ACS activity, stabilization of EILs and ERFs, and improved ethylene signaling, ultimately leading to enhanced salt tolerance. A recent study documented that under normal conditions, ETO1/EOL (components of CULLIN3 E3 ligase) proteins negatively affect ACS stability, maintaining low ethylene levels and suppressing autophagy. The SEVEN-IN-ABSENTIA of Arabidopsis (SINAT) is a RING-type E3 ligase family that may also negatively regulate the stability of ACS5 when stimuli induce their expression. During stress, BR induces phosphorylation in SINAT2 and ETO1/EOLs, recruiting 14-3-3 proteins, which activates the reciprocal degradation of the E3 ligases, in turn, increasing ACS5 abundance and autophagy activity by reducing SINAT stability [82]. Also, gibberellins are found to regulate the stability of the ACS5 protein [83]. In Arabidopsis, most *ACSs* are transcriptionally activated in response to auxin, and the spatial expression pattern of *ACS* is also modified upon auxin treatment [36]. A study reported that in 2-week-old light-grown Arabidopsis, *AtACS4* responds to IAA, ethylene, ABA, darkness, and wounding; *AtACS5* to IAA, ABA, salt, high temperature and wounding; and *AtACS7* to GA3, ethylene, ABA, darkness and salt. Each *AtACS* exhibits a distinct expression profile, suggesting continuous and stage-specific activity of the *AtACS* multigene family during Arabidopsis growth and development [84].

In addition, the homo- and heterodimerization of the ACS isoform influence enzyme activity and stability. Studies have revealed that the eight functional ACS proteins of Arabidopsis have the potential to form up to 45 different combinations of homodimers or heterodimers. However, due to structural constraints, only 25 of these combinations are functional and capable of forming active sites [66]. It was observed that ACS homodimers have enzymatic activity, while heterodimers consisting of ACS proteins from the same phylogenetic branch display activity [7]. According to a study, the stability of the labile ACS isoforms, ACS2 and ACS6, is notably prolonged when they form heterodimers with ACS7 instead of their respective homodimers. Notably, ACS7 stability is not affected by heteromerization with other ACSs. This suggests that the expression patterns of ACS7 may aid in regulating ACS turnover mediated by homodimerization or heterodimerization in Arabidopsis [79].

## 3. ACC Homeostasis and Its Signaling Function

Studies have shed light on the intricate regulatory mechanisms surrounding ACC, a key substrate in the ethylene biosynthesis pathway. ACC, generated by the catalytic activity of ACS, not only serves as a precursor for ethylene production but also exhibits a regulatory role independent of its conversion to ethylene [85]. The ACC pool undergoes strict regulation to serve both ACC signaling and ethylene biosynthesis. ACC conjugation plays a pivotal role in modulating the extent of ethylene biosynthesis; ACC showed the capacity for conjugation with 1-malonyl-ACC (MACC), γ-glutamyl-ACC (GACC), and jasmonyl-ACC (JA-ACC) [7,85]. The regulation of ACC involves not only conjugation but also transport, which plays a crucial role in controlling the spatial distribution of ethylene biosynthesis. Both short- and long-distance transport of ACC has been observed, with the xylem likely mediating the major transport route, and transportation via the phloem is also documented. Additionally, the intracellular transport of ACC across the transport into the vacuole has been demonstrated [7]. The identification of ACC transporters, such as LYSINE HISTIDINE TRANSPORTER1 (LHT1) and LHT2, has provided insights into the molecular mechanisms of ACC transport [86,87]. The discovery of novel ACC transporters is contingent upon further revelations in ACC mobility and homeostasis. Mounting evidence supports ACC’s role as a distinct signaling molecule beyond its established function in ethylene biosynthesis [86]. For instance, ACC regulates cell wall function via the FEI pathway. FEI1 and FEI2, LRR-RLKs linked to cellulose biosynthesis, impact root swelling and cellulose production. Notably, inhibiting ethylene biosynthesis reverses *fei1 fei2* mutants’ root phenotype, while ethylene perception blockage has no effect. Furthermore, the FEI kinase domain directly interacts with type 2 ACS proteins but not with type 1 and type 3. These results imply that FEI proteins define a novel signaling pathway that regulates cell wall function and that ACC is acting as a signaling molecule in this pathway [88]. The genetic study of Arabidopsis *ACS* mutants provides evidence supporting ACC’s role as a signaling molecule. An octuple mutant with a significant decrease in ethylene production displayed embryonic/gametophytic lethality and unfertilized ovules, whereas mutants completely insensitive to ethylene were viable. This outcome indicates that ACC, particularly ACS, possesses a function separate from the established ethylene response pathway. The disruption of the ACS gene family reinforces the diverse roles of its members in plant growth and development, highlighting both overlapping and unique functions [89]. Also, ACC has been observed to enhance the expression of genes associated with antioxidant defense mechanisms, safeguarding photosynthesis and respiration in seaweed during heat stress, indicating its role as a signaling molecule mitigating heat stress effects independently of ethylene [90]. Additionally, ACC promotes the development of sexual cells and protects *Pyropia* gametophytes from oxidative stress [91]. In the context of copper stress, ACC inhibits the expression of genes encoding Cu transport proteins, reducing copper accumulation, MDA contents, and mitigating growth inhibition in *G. lemaneiformis*, while also stimulating jasmonic acid synthesis and activating pathways unrelated to ethylene signaling [92]. Loss of ACS2 activity intensifies NaCl-induced inhibition of root growth, with the mechanism involving ACC accumulation activating IAA conjugases GH3.5 and GH3.9, ultimately leading to decreased IAA levels and impaired root growth [93]. A recent study showed that the dipeptide of ACC causes ethylene responses by means of substrate promiscuity mediated by ACO [94].

## 4. Structure and Regulation of ACO

### 4.1. ACO

The enzyme responsible for the final conversion of ACC to ethylene is 1-aminocyclopropane-1-carboxylic acid oxidase (ACO). ACO is classified within the 2-oxoglutarate-dependent dioxygenase (2OGD) superfamily, comprising non-heme iron proteins [95]. Few studies suggest that the 2-oxoglutarate-dependent dioxygenase enzyme is located in the cytosol, but others postulated that ACO is localized in the plasma membrane [78]. Based on similarities in their amino acid sequences, the three DOXA, DOXB, and DOXC subclasses of the plant 2OGD superfamily can be distinguished; ACO belongs to the DOXC subclass [95]. The 2OGD superfamily exhibits a distinct structural feature consisting of double-strand β-helix core folds, which commonly include a conserved 2-His-1 carboxylate motif derived from an aspartate or glutamate residue. This motif plays a vital role in the binding of Fe (II) at the enzyme’s catalytic site, facilitating the interaction with ACC. ACO functions as a reductant, triggering the opening of the ACC ring through its catalytic activity [96]. The amino group of ACC is coordinated to H177, and the carboxylate group of ACC is coordinated to D179, both of which are essential residues in the reaction center of ACO, making iron a prerequisite for the ACC binding mechanism [96]. Acting as a reducing agent, the ascorbate cofactor initiates the opening of the ACC ring [96]. Molecular oxygen and bicarbonate are activators in the ACO reaction mechanism, which speeds up the conversion of ACC to ethylene [97]. The main chain of the monomeric ACO enzyme consists of a secondary structure, i.e., 11 α-helices and 13 β-strands. Out of them, eight β-strands (4–11) form a distorted double-stranded β-helix common to all 2OGs. Most of the α-helices are located at the terminal end, α-1 to 6 at the N terminal. In contrast, α-8 to 11 at the C-terminus, in addition to this C-terminus, also has β-13, 310 helix, and two longer helices formed of α-9 and α-10,11, respectively [96]. Phylogenetic analysis suggests that the three types of ACOs originated simultaneously from a common ACO or 2ODG ancestor found in non-seed plants [9]. The H177-D179-H234 motif is necessary for ACO activity; however, residues R175, R299, and K158 have been proposed to play essential roles in coordinating the binding of bicarbonate [96,98], while residues K292, K158, and F300 are suggested as binding sites for ascorbate [98]. Furthermore, two highly conserved residues, R244 and S246, forming part of the RXS motif, contribute to the ACC/bicarbonate/ascorbate binding site in ACO [9]. Interestingly, the three types of ACO can be classified according to the intermediate residue found within the conserved RXS motif. Type I ACOs have an RMS intermediate residue, while type II ACOs and type III ACOs have an R-L/I-S intermediate and RRS intermediate residues, respectively. Dilley et al. [98] identified important residues for ACO activity. These residues are conserved in all three types of ACO, except for E294, E297, and E301. Among the three forms of ACO, E294 is not conserved, E297 is solely replaced by glycine in type II ACOs, and E301 is not conserved in type III ACOs. Future investigations are required to explore whether these variations in residue composition among the three ACO types correspond to differences in functionality, including enzyme activity and protein stability.

### 4.2. Transcriptional and Post-Transcriptional Regulation of ACO

Multiple transcriptional regulators of the *ACO* gene have been isolated from various species [7]. Lin et al. [99] reported that the first transcription factor found to control the expression of *SIACO1* directly was the tomato HD-ZIP (Homeodomain- leucine zipper) transcription factor SIHB-1. It was assumed that HB-1 might also regulate the ripening-related genes *PG1*, *RIN*, *NOR*, and *ACO2*. According to a subsequent study, the ripening regulator RIN may increase the expression of *HB-1* and *SIACO4* [100]. A study demonstrated that MdSnRK2 superfamily I protein kinases, by phosphorylating transcription factors MdHB1 and MdHB2, enhance the protein stability and transcriptional activity of *MdACO1* in apples [101]. NAC transcription factors SNAC4 and SNAC9 interact with *ACS2*, *ACS4*, and *ACO1* promoters, affecting tomato fruit ripening [102]. Reducing the expression of *SNAC4* and *SNAC9* by silencing these genes inhibits fruit maturation. In addition, suppressing *ACS4*, *ACO1*, and *ERF2* decreases the expression level of both *SNAC9* and *SNAC4*, indicating the presence of a tightly regulated feedback mechanism [102]. Wang et al. [35] reported that the acetylsalicylic acid (ASA)-responsive transcription factors AdERF105L and AdWRKY29 upregulate the expression of *AdACO5* in kiwifruit. In addition, ASA, by downregulating *AdAP* (aspartic peptidase), influences ethylene biosynthesis post-transcriptionally. A recent study documented that MaNAC083 downregulates *MaACO1/4/5*, thus reducing ethylene production during fruit ripening [103]. However, the documentation of transcription factors regulating *ACO* expression under abiotic stress is limited, necessitating further research. Some relevant findings are included in Table 1.

Recently discovered miRNA and *miR1917* were found to modulate the expression of *ACO* and the ethylene response in tomatoes by cleaving a CTR4 splice variant. The overexpression of *miR1917* substantially increased the level of the expression of *ACO1* and *ACO3*, resulting in ethylene response phenotypes, including triple response, leaf petiole curvature, pedicel abscission rate, and fruit maturation [48]. In the citrus variety *Poncirus trifoliata*, the cold-responsive *miR396b* enhanced cold tolerance in lemons when overexpressed, leading to reduced *ACO* expression relative to wild-type lemons [104]. A study showed that in Arabidopsis, *miR3933* post-transcriptionally cleaved the *ACO5*; similarly, in rice, *miR5809* and *miR531a/b* regulate *ACO* post-transcriptionally [50]. *VcMIR156a/VcSPL12* influences fruit color change in blueberries by regulating ethylene production through the targeting of *VcACS1* and *VcACO6*, demonstrating a crucial connection between the miR156/SPL regulatory module and the ethylene pathway [105].

### 4.3. Post-Translational Regulation of ACO

Post-translational modifications such as phosphorylation and glycosylation sites were supposed to be present in ACO by some researchers [98], but still, no study confirmed it. However, redox-specific changes have been identified in ACO proteins. For instance, in Arabidopsis, *AtACO2* was found to be targeted for S-glutathionylation at the C63 (Cysteine residue) target residue under stress conditions, confirming the post-translational modification by glutathionylation [41,106]. In addition, reports showed that ACO can undergo thiol-residue modifications known as sulfhydration. A study on tomatoes demonstrated that S-sulfhydration at C60 of *SlACO1* and *SlACO2* reduced ACO enzyme activity under osmotic stress [21]. Generally, thiol-group modifications like S-glutathionylation and S-sulfhydration play a role in protecting proteins from oxidation and redox changes as well as modulating protein–protein interactions [9]. Other post-translational modifications observed are S-sulfenylation and S-nitrosylation at C168 [7]. In petunia, the protein GRL2 (Green-like 2) directly binds to ACO1 and negatively inhibits its activity [107]. The precise impact of these modifications on ACO activity, protein interactions, and stability requires further investigation.

## 5. ACS and ACO in Relation to Abiotic Stress

In response to abiotic stress, ACS and ACO enzymes are modulated to regulate ethylene production, acting as mediators of stress adaptation. Both calcium-CDPK and MAPK signaling cascades are simultaneously activated, and their partial convergence contributes to the development of specific responses to each stimulus [78]. Table 2 summarizes how the expression of various *ACS* and *ACO* isoforms in plants is altered in response to different abiotic stress.

### 5.1. Heat Stress

An increase in temperature above the threshold level negatively affects cellular structures, physiology, and molecular function, thus reducing the growth and yield of plants [122]. Studies suggest that the involvement of ethylene in thermotolerance is through mitigating oxidative stress by inducing antioxidant defense machinery and maintaining the integrity and stability of plant cells [122,140]. In addition, the complex signal transduction network for thermotolerance includes genes related to ethylene synthesis and signaling, as well as heat shock proteins (HSPs) [141]. Notably, the EIN3-ERF95/ERF97-HSFA2 transcriptional cascade appears significant in the heat stress response, establishing a link between ethylene and its regulatory effects on plant thermotolerance [142]. ACC application to heat-exposed rice seedlings showed enhanced expression of HSPs and ethylene biosynthesis genes *ACO1* and *ACO3*, leading to reduced cell damage due to reduced H_2_O_2_ content than untreated seedlings [141]. Additionally, exogenous application of ethylene reduces the expression level of *ACS* and *ACO*, thus limiting ethylene stress, leading to enhanced photosynthesis, carbohydrate metabolism, and antioxidant defense and thermotolerance in *Oryza sativa* [140]. These contrasting findings highlight the complexity of ethylene’s role in plant stress responses. It is crucial to consider the specific experimental conditions; the effect of ethylene on heat tolerance may vary depending on factors, like the timing, duration, and intensity of heat stress. A study on tomato pollen grains exposed to heat stress revealed that under such conditions, the main *ACSs* responsible for ethylene production in pollen were identified as *SlACS3* and *SlACS11*. Moreover, during the mature pollen stage, heat stress resulted in the upregulation of *SlACO5* and downregulation of *SlACO4* in the anther wall [122], suggesting distinct expression patterns of multiple *SlACS* and *SlACO* during male reproductive tissue development and in response to heat stress. This diversity in gene expression potentially contributes to maintaining ethylene homeostasis, enabling precise regulation. Such intricate regulation is crucial because ethylene can have varying effects at different stages of plant development. Similarly, heat stress resulted in the upregulation of genes encoding *PsACS* and *PsACO* in pre-pollinated ovaries, leading to increased ethylene evolution in *Pisum sativum* plants. Furthermore, the study proposed that the expression of *ACO2* is closely associated with ethylene generation, as alterations in its activity affected ethylene production in various reproductive organs under heat stress [135]. In mango plants exposed to heat at 38 °C for three days, the generation of ethylene was suppressed due to the inhibition of both ACS and ACO. While ACO activity completely recovers after the heat treatment, ACS activity only partially recovers, and this partial recovery is adequate to enable heated fruits to attain an ethylene peak during ripening [143]. Silico analysis of ethylene biosynthesis genes in Arabidopsis under high-temperature stress showed that *ACS6*, *ACS7*, *ACS8*, *ACS10*, *ACS11*, *ACS12*, and *ACO2* were significantly upregulated, while *ACS2*, *ACS4*, *ACS5*, *ACO1*, *ACO3*, and *ACO4* were downregulated [4]. In rice, comparing heat-sensitive and heat-tolerant lines using transcriptomic data revealed the upregulation of *OsACS2*, *OsACS6*, *OsACO5*, and *OsACO7* in both lines under heat stress, whereas *OsACO1* and *OsACO2* were downregulated [4]. Genome-wide analysis of *Gossypium* species documented that *GhACS10.2* showed high expression at the early stage of heat stress; also, *GhACS12.2* responded to heat but *GhACS12.1* did not change under stress [116]. Upstream sequence analysis of GhACOs showed heat stress response elements (STREs) in cis-acting elements of these genes, confirming their role in heat stress [144]. In *Triticum*, under heat stress, the expression of *TaACS6* and *TaACS8* was increased consistently after 2 h of treatment; however, *TaACS3* and *TaACS10* were upregulated only after 24 h of treatment [49]. A recent study investigated ethylene biosynthesis in the heat-tolerant tomato cultivar “savior”, grown in winter and summer conditions. Gene expression analysis indicated higher ACO and ACS expression in winter, possibly influenced by heat stress affecting housekeeping genes. Despite seasonal variations, protein concentrations remained consistent, suggesting that heat stress did not impact ethylene biosynthesis-related protein abundance in this heat-tolerant cultivar. Enzymatic activity and proteomic analysis indicated that ACO5 and ACO6 isoforms, rather than ACO1, predominantly contributed to ACO activity in both winter and summer fruit [145]. Ethylene can modulate antioxidant enzymes, thereby influencing ROS metabolism [146]. The role of GSH (reduced glutathione) and ethylene was reported in terms of heat tolerance in a study by Rasheed et al. [147]. In addition, GSH induces ethylene formation by modulating ACS and ACO, both at transcriptional and post-transcriptional levels. Transgenic Arabidopsis plants (*AtECS*) with enhanced GSH content showed the upregulation of *ACS2*, *ACS6*, and *ACO1* at the transcript as well as protein levels, while the GSH-depleted *phytoalexin deficient2-1* (*pad2-1*) mutant showed downregulation of these genes responsible for the synthesis of key ethylene biosynthesis enzymes [41]. Though work on ethylene’s role in heat stress tolerance is reported through various studies, we find less evidence on how heat stress affects ACS and ACO isoforms at transcriptomics and proteomics levels. It is imperative to study the consequent changes in ACS and ACO under heat stress to modify the ethylene biosynthesis level at the gene level.

### 5.2. Heavy Metal Stress

Heavy metals (HMs) regulate plant metabolism and overall development at low concentrations, but excessive levels lead to cellular damage and toxicity. The displacement of essential metal ions or blocking of an essential functional group causes the inactivation of biomolecules and important pathways, causing heavy metal toxicity. This leads to the overproduction of ROS, degrading proteins and membranes. Studies show that HMs stimulate high-ethylene synthesis and play a convincing role in mitigating metal toxicity. For instance, on exposure to cadmium (Cd), chromium (Cr), copper (Cu), nickel (Ni), and zinc (Zn), increased ethylene production has been documented in many plants [148]. For instance, 5–500 µM concentration of Cu enhanced ethylene production in leaves of *Populus alba*, whereas Cu application at 25–50 µM in Arabidopsis showed no considerable increase in ethylene production [149]. The increase in ethylene evolution was due to the enhanced expression level of the ethylene biosynthesis enzymes *ACS* and *ACO* [110]. Transcriptomic analysis showed the enhanced expression of genes *OsACS1*, *OsACS2*, *OsACO4*, and *OsACO5* in rice roots treated with Cr, indicating that ethylene was involved in Cr signaling in rice [131]. The stability of ACS2 and ACS6 enzymes has been shown to increase through phosphorylation by MPK3 and MPK6 in Arabidopsis [53,54]. EIN2 is crucial in the ethylene signaling pathway and is suggested to play a role in response to Pb toxicity by regulating AtPDR12, an ABC membrane transporter responsible for removing Pb and Pb-containing toxic substances from the cytoplasm [150,151]. The involvement of MAPKs in mediating signaling responses in plants exposed to metal stress implies a potential influence on ethylene biosynthesis. A report demonstrated a sequential increase in the expression level of *StACS5* and *StACS4* in potatoes under Cu exposure, indicating that both genes have different signal transduction and gene regulatory mechanisms [152]. Additionally, Cu has been shown to increase the expression of *NgACO1* and *NgACO3* in *Nicotiana glutinosa*, indicating enhanced ethylene production [153]. It has been observed that Cd is potentially the most phytotoxic, capable of inducing ethylene production in many plants, like Arabidopsis, *B. juncea*, and *H. vulgare*, and this has been well documented [148]. A report suggests ethylene negatively regulates the suberization of endodermis under HM stress. In *Sedum alfredii* exposed to Cd, endogenous ethylene production was reduced in the high-accumulating ecotype (HE) by suppressing *SaACS2*, *SaACS6*, and *SaACO2*. Conversely, the non-high-accumulating ecotype (NHE) exhibited increased ethylene production under Cd stress. However, HE consistently had higher ethylene emissions than NHE due to the continual higher expression of *SaACS2*, *SaACS6*, and *SaACO2*, regardless of Cd concentration. The elevated levels of ethylene in HE delayed the formation of apoplastic barriers by inhibiting phenylalanine ammonia-lyase activity and gene expression of lignin and suberin biosynthesis, leading to increased Cd accumulation in the root apoplast [154]. Cadmium triggered the synthesis of ACC and ethylene in *Arabidopsis thaliana* plants, primarily through upregulating *ACS2* and *ACS6* expression. This relationship was validated by studying the *acs2-1acs6-1* double-knockout mutants, which exhibited reduced ethylene production. As a result, these mutants displayed enhanced leaf biomass and experienced delayed activation of ethylene-responsive genes, with no significant variations in Cd levels between the wild-type and mutant plants [110]. A recent study found metal response element (AP-1 and O2-site) cis-acting elements in *ACO* genes of *Gossypium*, implying its role in metal stress [144]. Khan and Khan [155] reported that under Ni and Zn toxicity, ACS activity and ethylene evolution increase; however, ethephon (ethylene donor) application under stress was found to reduce the ACS activity and bring ethylene production to the optimum level, thus enhancing antioxidant machinery and improving the PSII (photosystem II) efficiency, N-use efficiency, and photosynthesis in mustard plants. Similarly, under Cd toxicity, applying gibberellic acid plus sulfur reduced the ACS activity and limited the stress ethylene to a range suitable for promoting GSH production, sulfur-use efficiency, and photosynthesis in mustard [156]. Studies showed that the application of ethephon reduced the HM-mediated oxidative stress and protected the photosynthetic capacity through enhanced antioxidant defense machinery by maintaining optimum ethylene levels, whereas the higher concentration showed a negative effect on plant growth [155,156]. This revealed the complex and biphasic regulatory function of ethylene under stress conditions. ACS and ACO enzymes play critical roles in mitigating heavy metal stress by promoting ethylene production, which activates stress-responsive genes. However, the specific transcription factors governing ACS and ACO isoform regulation under heavy metal stress remain poorly understood.

### 5.3. Drought Stress

With climate change, a continuous change in atmospheric conditions leads to global warming and sub-normal rainfall over longer periods, affecting the groundwater level [157]. An increase in average temperature enhances water evaporation, leading to drought conditions, and this water scarcity leads to multiple unfavorable consequences on the productivity of plants, resulting from abnormal physiological processes, such as reduced turgidity, water potential, carbon assimilation rate, gaseous exchange, and overproduction of ROS, causing oxidative damage [157]. To cope with these challenges, plants adopt different strategies, such as drought-induced ethylene production causing leaf abscission as a mechanism to conserve water [158]. When plants experience a water deficit, ethylene production coincides with a rise and subsequent decline in ACC levels. Analysis of *Glycine max* plants, tolerant to water stress, showed differential expression of *ACS*, *ACO*, *ETR*, and *CTR*. It was found that the expression of ethylene biosynthesis genes *ACS* and *ACO* was upregulated, while the expression of *CTR* (ethylene signaling component) was downregulated, indicating the involvement of ethylene biosynthesis and signaling pathways in soybeans’ response to water stress [118]. Similarly, the mutants with edited versions of *PhACO1* and *PhACO3* in petunia exhibited reduced ethylene production and increased sensitivity to drought stress [134]. Molecular analysis revealed significant differences in the expression levels of genes related to antioxidant activity, proline synthesis, ABA synthesis and signaling, and ethylene signaling between the wild-type and mutants, suggesting the involvement of ethylene in the transcriptional regulation of genes associated with tolerance to abiotic stress [134]. A report by Du et al. [129] demonstrated that *OsETOL1* (ETHYLENE OVERPRODUCER 1-LIKE) overexpression reduced ethylene, making plants drought-susceptible; OsETOL1 interacted with OsACS2, inhibiting its activity, thereby reducing ethylene production. Thus, this can lead to a decrease in grain filling and spikelet fertility and a delay in the maturation process induced by ethylene.

The transcription factor OsERF109 negatively affects drought resistance in rice as overexpressing (OE) plants lose water faster, while RNA-interfering (RI) plants resist drought better. OsERF109 influences ethylene production, with OE lines producing less ethylene due to reduced *OsACS6* and *OsACO2* expression and RI lines producing more ethylene with increased *OsACS6* and *OsACO2* expression, affirming ethylene’s positive role in drought tolerance through the regulation of its biosynthesis enzymes, also overexpression of OsERF3 results in reduction of ethylene production and sensitivity to drought by suppressing expression of *OsACS2/6* and *OsACO2/3* [44,159]. The study investigated the impact of water deficit on the gene expression of *ACS* and *ACO* in various plant organs of *Coffea arabica*. Notably, *CaACS7* in leaves showed the most significant reduction in expression, while *CaACS1* in roots was induced during water deficit, possibly leading to increased ACC levels. *ACOs* were generally repressed in roots during drought, with *CaACO4* being the most affected. This repression of *ACO* expression led to an accumulation of ACC in roots, crucial for triggering anthesis once plants are rehydrated. The subsequent return of rain or irrigation means the ACCs were transported to the leaves and converted to ethylene by ACO enzymes [160]. A genome-wide study on cotton species showed that most of the *GhACS6* responded to drought stress at the early stage, whereas the expression of *GhACS6.1*, *GhACS10.2*, and *GhACS12.1* did not change [116]. In addition, at upstream sequences of the promoter of *GhACOs*, various drought response elements, like DRE core, DRE1, MYC, MBS, and MYB recognition sites, are observed, indicating its role in drought stress [144]. Furthermore, wheat genome-wide analysis showed that the expression of *TaACS3/6/10* was upregulated, and among them, *TaACS3/10* was induced within 1 h after treatment, while the expression of *TaACS8* was downregulated [49]. Transcriptomic analysis shows that MaDREB1F influences the genes responsible for producing ethylene directly or indirectly through MaERF11, triggering the expression of *MaACO20* [161]. The targeting of *TaACS11* by *tae-miR531* implies that *TaACS11* could be influenced in its response to drought stress through this specific regulatory mechanism [49]. These reports indicated that drought stress induced ethylene production, thus showing that some factors regulate the activity and expression of ethylene biosynthesis enzymes ACS and ACO, influencing various physiological and molecular processes in plants.

### 5.4. Salinity

Studies have observed the induction of *AtACS7*, *AtACS5*, and *NtACSO3* in Arabidopsis and tobacco plants under salt stress conditions [128,162]. Furthermore, in Arabidopsis, it was found that *AtACS2*, *AtACS6*, *AtACS7*, and *AtACS8* were triggered by high salt stress, while moderate to low salinity (salt acclimation) mitigated this induction. Furthermore, in both non-acclimated and salt-acclimated stress conditions, these genes exhibited upregulation, indicating the essential role of ethylene production in facilitating plant adaptation to challenging environments [163]. In a recent study, it was found that under salt, stress expression and the activity of ACS increase, leading to high ethylene production called stress ethylene affecting the photosynthesis of wheat plants, while melatonin application reduced ethylene to the optimal level by modulating *ACS* expression and activity, leading to resistance to salinity [164]. A comprehensive investigation demonstrated that the stress-triggered MAPK pathway could potentially trigger the activation of WRKY33, subsequently stimulating the upregulation of *ACS2/ACS6* in Arabidopsis [55]. The overexpression of *TaACO1* in transgenic Arabidopsis plants led to augmented transcription of AtMYB15, accompanied by the downregulation of *AtCBF1* and *AtCBF3*, resulting in increased susceptibility to salt [165]. Mutants with edited ethylene biosynthesis genes *phaco1* and *phaco3* exhibited differences in the expression levels of genes related to antioxidant defense, proline synthesis, ABA synthesis and signaling, and ethylene signaling, showing increased sensitivity to salt stress compared to wild-type plants of Petunia [134]. A recent study documented that the ACO homolog 4 (ACOh4) was crucial in regulating ethylene synthesis and salt tolerance through NO-mediated S-nitrosylation in the roots of tomato plants. Additionally, NO was found to induce the transcriptional expression of ACOh4. The knockdown of ACOh4 abolished NO-induced ethylene production and salt tolerance. The ACOh4 positively regulated Na^+^ and H^+^ efflux, maintained the K^+^/Na^+^ homeostasis, and promoted the transcription of salt resistance genes [166]. GSH via WRK33 showed upregulation of *AtACS2*, *AtACS6*, and *AtACO1* at the transcript and protein levels in Arabidopsis *AtECS1* mutants (plants with enhanced GSH content), providing resistance to salt stress [41]. Additionally, Ca^2+^ upregulated the expression of specific genes (*CsACS3*, *CsACO1*, and *CsACO2*) associated with ethylene production during adventitious rooting in *Cucumis sativus* under salt stress [167]. In *Gossypium hirsutum* leaves, short- and long-term salt stress resulted in the upregulation of *ACS1*, *ACS12*, *ACO1,* and *ACO3* [117]. In addition, a genome-wide study of the *ACS* gene in *Gossypium* species showed that after 6–12 h exposure to 400 mM NaCl, *GhACS1* was upregulated, while *GhACS2*, *GhACS6.1*, *GhACS6.2*, and *GhACS6.4* were downregulated [116]. The overexpression of the cotton plant gene *GhACO106* in Arabidopsis enhanced salinity tolerance [144]. Similarly, wheat genome-wide analysis showed that salt stress induces the expression of TaACS3/6/7/9/10, and the expression of *TaACS9* reached its maximum level after 24 h of salt stress. Also, it was reported that the cis-regulatory elements of the promoter of these genes contain DRE elements, indicating their role in stress [49]. Thus, ethylene production rises in response to stress, primarily due to increased ACS and ACO expression and activity, with variability based on plant species, organ, and concentration. Contrarily, the decreased transcript of *ACO1* has been reported in wheat under salinity and other abiotic stresses [165]. Similarly, Tao et al. [168] reported through various studies in their review that functional knockout of some ACSs increased plant’s ability to tolerate salinity, and the overexpression of *ACSs* increased salt sensitivity

### 5.5. Flooding

During flooding, the diffusion of gases between plant cells and the outside environment is restricted, causing hypoxic conditions that affect physiological processes such as photosynthesis and respiration. In complete submergence, ethylene synthesis increases and is entrapped in plant tissues [169]. It was documented that ethylene triggers adventitious root development, shoot growth towards the surface for gaseous exchange, and aids in metabolic acclimation to hypoxic conditions under submergence [169,170]. Levels of ethylene were significantly higher in submergence-intolerant (M202) genotypes compared to the tolerant (M202-*Sub1*) genotype of rice. This difference was regulated by suppressing the *ACS2* gene in the tolerant genotype [171]. In rice growing under low-oxygen conditions, the expression of *ACS1* and *ACS5* was found to be increased [12]. These genes were differentially expressed in various tissues during submergence. *ACS1* transcripts are predominantly in the cell elongation zone of the internode, whereas *ACS5* transcripts are expressed in regions characterized by extensive cell division and elongation, such as vascular bundles in leaf sheaths and young stems [172]. These studies proposed that *ACS5* and *ACS1* worked together to produce ethylene, leading to the elongation of internodes and increased cell division and elongation in the vascular bundles of young stems during extended submergence. Transcriptome analysis revealed the upregulation of *OsACO1* in the epidermal cells, located above the adventitious root primordia, indicating that enhanced ACO1 activity in this localized region could enable the precise spatial regulation of ethylene biosynthesis [173]. Yamauchi et al. [170] proposed that when rice seedlings were subjected to stagnant flooding and provided with adequate oxygen along with an ethylene inhibitor, the formation of aerenchyma, an important adaptation for flood tolerance, was suppressed. Furthermore, studies have reported a strong induction of *OsACO8* and *OsACO3* in submerged rice shoots, while *OsACO1* was negatively regulated. Further research has provided evidence indicating that *OsACO5* exhibited high expression levels under normal oxygen conditions, suggesting its primary role in the formation of aerenchyma in well-aerated roots of rice [130]. On the other hand, the expression of *ACO1* was accountable for the accumulation of ethylene following the initiation of aerenchyma formation. At the same time, *ACO5* played a crucial role in maintaining ethylene synthesis in the roots. The significant downregulation of *OsACO1* observed that in the tolerant genotype, M2O2-*Sub1* may be associated with ethylene-mediated submergence responses, including shoot elongation and the inhibition of carbohydrate utilization [130]. In contrast, studies also demonstrated the negative role or no role of ethylene in plant survival under submergence [174]. Therefore, gaining knowledge about ethylene production in plants that exhibit tolerance to flooding can improve food crop resilience.

## 6. ACS and ACO in Nutritional Deficiency

The imbalances in nutrient levels can significantly impact the performance of plants, resulting in modifications in a range of physiological processes, overall growth patterns, and the ability to withstand both biotic and abiotic stresses. Plants undergo various changes in their morphology and physiology to adapt to nutrient deficiencies [175]. Next, we outline some studies highlighting the connection of ethylene in the regulation of different nutrient deficiencies. The interplay between ethylene and nitrogen (N) availability influenced various physiological processes, encompassing the architecture of root, leaf, and reproductive organ development and the synthesis of amino acids, proteins, and enzymes [176]. A study reported that under N deficiency, endogenous ethylene evolution increased, referred to as stress ethylene, negatively affecting plants. However, under specific conditions where the perception of ethylene was enhanced, it has been observed that ethylene improved the nitrogen use efficiency and overall growth of plants grown under optimal and deficient N conditions [177]. Similarly, another study reported that ethylene concentration was higher in mustard plants grown at low N compared to controls. The application of exogenous ethylene further increased the ethylene evolution, increasing the nitrate reductase activity, total nitrogen content, photosynthesis, and plant growth [178]. The availability of N regulated the formation of ethylene by influencing ACS activity. This, in turn, governed the levels of N content and NR activity [179]. Furthermore, it has been shown by meta-profiling research that the ethylene biosynthesis pathway is regulated by N levels. For example, under nitrate deficiency, the expression of *ACS7* and *ACO10* was significantly repressed in seedlings. However, when leaf samples were exposed to either low or high N levels, both *ACS7* and *ACO10* were activated [176]. Conversely, *ACS8*, *ACS4*, *ACO1*, *ACO5*, and *ACO2* were downregulated under low- and high-N conditions. It is intriguing to consider that the families of *ACS* and *ACO* genes exhibited distinct temporal and spatial expression patterns in response to varying N levels [176]. During the initial response of cucumber seedlings to N starvation, a thorough analysis of the plant’s transcriptome revealed the presence of a homolog of ACO6, which was involved in ethylene synthesis [180]. Moreover, prolonged low-N conditions revealed the upregulation of an *ACO4* homolog and a transcript similar to *ACO* in response to N starvation [176]. A report demonstrated that in response to low-nitrate treatment, *Arabidopsis thaliana* (Col-0) seedlings exhibited a rapid burst of ethylene production, which may be due to increased activity or transcript levels of ACS and ACO, along with the altered expression of ethylene signaling components CTR1, EIN3, and EIL1. This low-nitrate treatment also increased the ethylene response reporter EBS/GUS activity in both Col-0 and ethylene mutants *ein3-1eil1-1* and *ctr1-1.* Additionally, the expression of *NRT2.1*, a nitrate transporter involved in high-affinity nitrate uptake, was upregulated upon low-nitrate treatment. Comparisons between *nrt1.1* and *nrt2.1* mutants and Col-0 indicated that the increased *NRT2.1* expression positively affected ethylene biosynthesis and signaling in low-nitrate conditions. In contrast, ethylene suppressed the expression of *NRT2.1*, which decreased the absorption of high-affinity nitrate. These results demonstrated the existence of a negative feedback loop, linking *NRT2.1* expression with both ethylene biosynthesis and signaling during nitrate deprivation. This feedback loop could help to precisely control how plants acquire nitrate in response to changing soil conditions [181]. Tian et al. [182] reported that high nitrate levels stimulated ethylene production in roots by activating *ACS* and *ACO*. They also showed the regulatory role of ethylene in nitrate-dependent root development through the modulation of nitrate transporters *NRT1.1* and *NRT2.1*, thereby influencing nitrate uptake and root elongation.

The phosphorus (P) deficiency, leading to ethylene generation or alterations in ethylene sensitivity, has played a crucial role in regulating the root architecture and the root hydraulic conductivity [183]. Ethylene has been shown to govern the expression of *PSI* (*PHOSPHORUS STARVATION INDUCED*), participate in Pi transport, signaling, and recycling, and stimulate the activity of APases (acid phosphatases; intracellular APases participate in Pi remobilization within plants, while secreted APases contribute to Pi release from organophosphates in the rhizosphere, enhancing Pi availability for root uptake) but suppress anthocyanin accumulation during phosphorus deficiency [184]. Also, ethylene exerts its regulatory influence at the transcriptional and post-transcriptional levels to modulate plant P responses. In response to P deficiency, it was documented that EIN3/EIL1 regulated the PHOSPHATE STARVATION RESPONSE 1 (PHR1) transcription factor, involved in stimulating the expression of genes involved in P acquisition, such as *PAP17*, *PHT1;1*, and *PHT1;4* [185]. EIN3/EIL1 through the WRKY75 transcription factor was involved in the expression of the internal phosphate transporter *PHT1;5* [186]. However, there are limited reports regarding the specific ACS and ACO isoforms that are differentially regulated in these specific responses. Microarrays and RNA-seq analyses have confirmed the upregulation of ethylene biosynthetic genes, particularly *ACS* and *ACO*, in Arabidopsis experiencing Pi starvation [184]. A study demonstrated that the expression of *ACS2*, *ACS4*, and *ACS6* increased in Arabidopsis seedlings when grown under Pi-deficiency conditions [187]. It was observed that the elevated expression of *ACS2* and *ACS6* reverted to normal levels when Pi-deficient plants were supplied with adequate Pi, indicating a cause–effect interaction between the expression of these genes and the Pi levels in the environment [188]. In the *phr1phl1* (*phosphorus starvation response*; *phr1-like1*) double mutant of Arabidopsis, the activation of several *PSIs*, including *ACS6* and *ACS7*, was hindered to varying degrees. Notably, the expression of two other *PSIs*, *ACS2* and *ACS4*, was not dependent on the PHR1 pathway [189]. This indicated that different signaling pathways mediated the regulation of other members of the *ACS* family. The response of *ACS* and *ACO* families to Pi deficiency differs among plant species. Studying the transcription of specific *ACS* and *ACO* in different tissues and developmental stages under Pi deficiency is crucial for understanding the spatiotemporal regulation of ethylene biosynthesis.

A microarray experiment on Arabidopsis showed that under potassium (K^+^) deficiency, the expression of genes *ACS* and *ETR2* and reactive oxygen production increased. In downstream signaling, ethylene enhanced the expression of *HIGH-AFFINITY K^+^ TRANSPORTER 5* (*HAK5*), which may be through RAP2.11, an ethylene response factor [190]. This was confirmed by using ethylene-insensitive mutants *ein2-1*, where a reduction in *HAK5* expression was observed [190]. In addition, K deprivation inhibited lateral root growth but stimulated root hair growth in Arabidopsis. This response was attributed to increased ethylene production in low conditions [191]. A direct study of ACS and ACO’ role in regulating K deficiency still needs to be conducted.

Under magnesium (Mg) deficiency, the upregulation of *ACS11* was observed in both roots and leaves, whereas *ACS2*, *ACS7*, and *ACS8* were specifically upregulated only in the leaves of Arabidopsis [192]. A recent study revealed that under magnesium-deficient conditions, the promotion of root hair elongation in Arabidopsis was facilitated through the increase in ethylene biosynthesis (ACS7 and ACO1) and signaling (ETR1) via the *KAI2-KL* (*KARRIKIN INSENSITIVE2*) signaling pathway [193]. Another study revealed that the interaction between ethylene and NO effectively governed the root hair development under conditions of Mg deficit. Ethylene stimulates NO generation through the NR (nitrate reductase) and NOS-L (nitric oxide synthase-like) pathways. At the same time, NO enhances the release of ethylene by activating ACO and ACS enzymes. Consequently, a positive feedback loop between NO and ethylene was established, thereby regulating the growth of root hairs in response to Mg deficiency in Arabidopsis [194].

Plants can be classified into Strategy I and Strategy II groups based on their iron (Fe) uptake mechanisms. Strategy I plants convert Fe^3+^ to Fe^2+^ before uptake, while Strategy II plants excrete phytosiderophores (PS) to chelate Fe^3+^ for uptake [186]. Ethylene regulated the expression of *FIT* (FER-*LIKE Fe DEFICIENCY-INDUCED TRANSCRIPTION FACTOR*), *FRO2* (*FERRIC REDUCTASE OXIDASE*), *IRT1* (*IRON REGULATOR TRANSPORTER*), *NAS1/2* (*NICOTIANAMINE SYNTHASE*), and flavin synthesis genes, enhancing ferric reductase activity and Fe^2+^ uptake [186]. In Strategy II plants, ethylene activates transcription factors IDEF1 and IRO2, leading to the upregulation of *NAS* for PS (phytosiderophores) synthesis and expression of PS-Fe^3+^ transporter YSL15 for iron acquisition [195]. In Strategy I species and rice, it was reported that Fe deficiency upregulated the ACS and ACO for ethylene formation [196,197]. Fe deficiency upregulated *AtSAM1*, *AtSAM2*, *AtACS4*, *AtACS6*, *AtACS9*, *AtACO1*, and *AtACO2* (ethylene synthesis) and AtETR1, AtCTR1, AtEIN2, AtEIN3, AtEIL1, and AtEIL3 (ethylene signaling) in the roots of Arabidopsis for iron acquisition and homeostasis [197]. Under iron-deficiency conditions, both the expression and activity of MPK3/MPK6 are heightened, leading to increased transcript levels of ACS2 and ACS6. Research suggests that ACS stabilization is pivotal in sensing nutrient deficiency [198].

In context to sulfur (S), Iqbal et al. [199] documented that the application of ethephon enhanced the photosynthetic responses in two mustard cultivars that differed in photosynthetic capacity by increasing the ATP sulfurylase activity and enhanced the S content. This showed a connection between ethylene and S assimilation. In S-deficient *Brassica napus* plants, it was observed that ethylene, by regulating the expression of S transporter genes *BnSultr1,1*, *BnSultr1;2*, *BnSultr4;1*, and *BnSultr4;2,* influenced S acquisition [200]. Low-sulfur (LSU)-like proteins are reported to function as modulators of ethylene biosynthesis under S-deficiency conditions by affecting either the function or stability of the ethylene synthesis enzymes. ACS or ACO serves as the key enzyme in the regulation of this pathway [27,74]. LSU-like proteins directly interact with ACO to facilitate ethylene production when plants are exposed to S deficiency [201].

Under boron (B) deficiency, an increase in *ACS11* expression led to elevated levels of ACC and ethylene, resulting in altered auxin response and reduced cell elongation in the primary root of Arabidopsis seedlings, while root hair formation and length increased [202]. A recent report suggested that under B deficiency, cytokinins stimulated the expression of *ACS11*, thus inhibiting root cell elongation in an ethylene-dependent manner. In addition, the ethylene-independent pathway was also present where cytokinins downregulated *AUX1*, leading to altered auxin signaling in the meristematic and maturation zone, causing the reduced elongation of root cells in Arabidopsis [203].

In the context of calcium (Ca), it was reported that calcium plays a pivotal role in interacting with ethylene signaling and governing plant responses. Calcium is essential for the induction of genes and the proper functioning of ethylene receptors. Calcium deficiency impairs ethylene-related responses, while increasing Ca levels enhances the responses [204]. The application of verapamil (Ca^2+^ inhibitor) promoted ethylene production by upregulating the expression of *LeACO1* and downregulating *LeETR4*. At the same time, Ca treatment delayed the commencement of and maximum ethylene production, maintaining low *LeACO1,* but raised the expression of *LeETR2/3/4/5*. The study suggested that Ca was involved in preventing ethylene production during the conversion of ACC to ethylene, and it regulated the expression of ethylene receptors [205]. According to a recent study, in treatments with 10 µM calcium chloride, Ca^2+^ enhanced ACS and ACO activities, promoted ACC and ethylene production, and regulated the expression of genes involved in ethylene signaling [167].

Taken together, the existing research highlights the involvement of ethylene in regulating plant responses to nutrient deficiencies. However, a comprehensive understanding of how nutrient deficiencies regulate ACS and ACO genes at the transcriptional, post-transcriptional, and translational levels, as well as the specific isoforms involved, is still a subject of ongoing scientific investigation. This knowledge is crucial for developing targeted strategies to improve nutrient utilization in plants, enhance crop yields, and mitigate the effects of nutrient deficiencies in agriculture.

Table 3 summarizes the differential expression of *ACS* and *ACO* in response to different nutritional stress.

## 7. Conclusions and Future Perspectives

Understanding the intricate regulatory mechanisms of ACS and ACO enzymes in ethylene biosynthesis is crucial for unraveling the complexities of plant development and stress responses. The advancements in research have significantly advanced our understanding of the molecular mechanisms regulating ACS and ACO. The modulation of ACS and ACO turnover has been critical in various processes, such as fruit ripening, senescence and abscission of leaves, flower development, and specific types of abiotic and biotic stresses. Experimental results showed that Arabidopsis corresponding to *ACS* and *ACO* genes in other plant species may have different regulatory mechanisms and functions, implying that different ACSs and ACOs may mediate abiotic stresses in various plant species, indicating a functional diversity of these genes in adapting to external stimuli. This knowledge opens avenues for developing innovative approaches to modulate ethylene signaling in plants, thereby enhancing their adaptability to challenging environments. However, further investigations are required to fully understand the precise mechanisms governing the regulation of ACS and ACO, especially regarding how abiotic stress influences their transcriptional, post-transcriptional, post-translational, and epigenetic modifications and regulates specific isoforms of ACS and ACO. ACS and ACO protein stability is influenced by various plant hormones, with different isoforms being affected differently; also, how nutrient deficiency modulates their expression, the specific components, and the mechanisms underlying this regulation remain largely unknown and need to be explored.

## Figures and Tables

**Table 1:** Transcription factors regulating ACS and ACO under abiotic stress.

S.No.	Plant	Transcription Factor	Gene Targeted	Up/Downregulated	Plant Organ	Plant Response under Abiotic Stress	Reference
**1.**	*Arabidopsis*	AtMYB30	*AtACS7*	Down	Root and Leaves	Ethylene synthesis reduced, leading to submergence tolerance	[39]
		EcAGL	*AtACS2*	Down	Leaves	Reduce ethylene synthesis, thus enhancing Cd-tolerance by inhibiting Cd-transport from root to shoot.	[40]
		AtWRKY33	*AtACS2*, *AtACS6*	Up	Leaves	Enhanced ethylene synthesis resulting in salt tolerance	[41]
		AtSHYG	*AtACO5*	Up	Leaf petiole	Enhanced ethylene synthesis leading to rapid petiole cell expansion and hyponastic leaf movement	[42]
**2.**	*Malus domestica*	MdERF1B	*MdACS1*, *MdACO1*	Up	Leaves	Enhanced ethylene production leading to cold tolerance	[43]
**3.**	*Oryza sativa*	ERF109	*ACS6*, *ACO2*	Down	Leaves	Reduce ethylene production and enhance drought tolerance	[44]
**4.**	*Poncirus trifoliata*	PtrERF9	*PtrACS1*	Up	Leaves	Feedback regulation of ethylene to orchestrate cold stress response	[33]
**5.**	*Triticum aestivum*	TaMYC8-TaERF6	*TaACS*, *TaACO*	Down	Root	Ethylene production decrease, leading to Cd-tolerance	[45]
		TabHLH094-TaMYC8	*TaACS*, *TaACO*	Down	Root and leaves	Ethylene synthesis decreases, leading to reduced uptake of Cd into roots	[46]

**Table 2:** Differential expression of various *ACS* and *ACO* isoforms in response to different abiotic stress.

S.No.	Plant	Target Genes	Up/Downregulated	Plant Organ	Type of Stress	Reference
**1.**	*Arabidopsis thaliana*	*ACS 2*,	Up	Leaves	Hypoxia	[108]
		*ACS9*, *ACS6*, *ACS7*	Up	Leaves and roots	Hypoxia	[108]
		*ACS6*	Up	Leaves	NaCl, LiCl, CuCl_2_	[109]
		*ACS2*, *ACS6*, *ACO2*, *ACO4*	Up	Leaves and roots	Cadmium	[110]
		*ACS11*	Up	Leaves	Salinity, cold, drought & flooding	[17]
		*ACS6*, *ACS7*, *ACS8*, *ACS10*, *ACS11*, *ACS12*, *ACO2*	Up	-*	Heat	[4]
		*ACS2*, *ACS4*, *ACS5*, *ACO1*, *ACO3*, *ACO4*	Down	-*	Heat	[4]
		*ACS2*, *ACS4*, *ACS8*	Down	Root tip	Anaerobiosis	[36]
		*ACS4*, *ACS5*, *ACS7*	Down	Root tip	Lithium treatment	[36]
		*ACS5*	Up	Root tip	Lithium treatment	[36]
	*Arabidopsis thaliana* (GM-OE-ACO)	*ACO*	Up	Leaves	Flooding	[111]
**2.**	*Agrostis stolonifera*	*ACO*	Up	Leaves	Cold	[112]
		*ACO*	Down	Leaves	Drought and NaCl	[112]
**3.**	*Chenopodium quinoa*	*ACS7a*, *ACS10a/b*, *ACS12a*	Up	Shoot	Heat	[113]
		*ACS6a/b*, *ACS7a*	Up	Root	Heat	[113]
		*ACS1b*, *ACS12a/b*, *ACS10*	Up	Shoot	Salt	[113]
		*ACS10a*, *ACS12a*	Down	Shoot	Salt	[113]
		*ACS10b*, *ACS12a*, *ACS10a*	Up	Root	Salt	[113]
		*ACS1a*	Down	Root	Salt	[113]
		*ACS6a/b*, *ACS7a*, *ACS9a*	Up	Root and leaves	salt	[113]
		*ACS1a/b*, *ACS6a/b*, *ACS7a*, *ACS9a*	Up	Root	Drought	[113]
		*ACS10a*, *ACS12a*	Up	Root and leaves	Drought	[113]
**4.**	*Cucumis sativus*	*ACS1*	Up	Fruit skin	Drought	[114]
		*ACS1*, *ACS2*, *ACS3*	Up	Leaves	Salt, drought, cold	[115]
		*ACO1*, *ACO2*	Up	Leaves	Salt, drought, cold	[115]
**5.**	*Gossypium hirsutum*	*ACS1*	Up	-*	Salt	[116]
		*ACS 2*, *ACS6.1*, *ACS6.2*, *ACS6.4*	Down	-*	Salt	[116]
		*ACS6.1*, *ACS6.3*, *ACS7.1*, *ACS10.1*, *ACS10.2*	Up	-*	Cold	[116]
		*ACS6.2*, *ACS12.2*	Up	-*	Heat	[116]
		*ACS1*, *ACS12*, *ACO1*, *ACO3*	Up	Leaves	Salt	[117]
**6.**	*Glycine max*	*ACS*, *ACO*	Up	Leaves and roots	Drought	[118]
**7.**	*Lycopersicon esculentum*	*ACS1*, *ACS2*, *ACS6*, *ACS7*, *ACO1*, *ACO2*, *ACO3*, *ACO5*	Down	Leaves	UV	[119]
		*ACS3*, *ACS5*	Up	Leaves	UV	[119]
		*ACS1*, *ACS7*, *ACO3*	Up	Roots	UV	[119]
		*ACS6*, *ACO1*	Down	Roots	UV	[119]
		*ACS2*, *ACS3*	Up	Roots	Flooding	[120]
		*ACS2*, *ACS6*, *ACO1*, *ACO3*	Up	Leaves	Ozone	[121]
		*ACO5*	Up	Anther wall [at mature pollen grain (MPG stage) of development]	Heat	[122]
		*ACS2*, *ACO4*	Down	Anther wall (MPG stage)	Heat	[122]
		*ACS3*, *ACS11*	Up	Pollen grain [at polarized microspore (PM) and Bicellular pollen grain (BCP) of development]	Heat	[122]
		*ACS4*, *ACO3*	Down	Pollen grain (at PM stage of development	Heat	[122]
		*ACO1*, *ACO4*	Down	Pollen grain (at BCP stage of development)	Heat	[122]
**8.**	*Malus acuminata*	*ACS1*, *ACO1*	Down	Fruit	Cold	[123]
**9.**	*Medicago sativa*	*ACS*, *ACO*	Up	Leaves	Waterlogging	[124]
**10.**	*Medicago truncatula*	*ACS2*, *ACO1*	Up	Leaves	Cold	[125]
**11.**	*Morus nigra*	*ACS1*, *ACS3*	Up	Leaves	Salt/drought	[126]
**12.**	*Morus alba*	*ACO1*	Up	Leaves	Cold	[127]
**13.**	*Nicotiana tabacum*	*ACO1*, *ACO2*, *ACO3*	Up	Leaves	Salt	[128]
		*ACS1*	Down	Leaves	Salt	[128]
**14.**	*Oryza sativa*	*ACS2*	Up	Leaves	Drought/submergence	[129]
		*ACS1*, *ACO5*	Up	Roots	Waterlogged	[130]
		*ACS5*	Up	Stem	Submergence	[12]
		*ACS1*	Down	Stem	Submergence	[12]
		*ACS1*, *ACS2*, *ACO4*, *ACO5*	Up	Roots	Cr-stress	[131]
		*ACS2*, *ACO4*	Up	Roots	As-stress	[132]
		*ACS2*, *ACS6*, *ACO5*, *ACO7*	Up	-*	Heat	[4]
		*ACO1*, *ACO2*	Down	-*	Heat	[4]
		*ACS1*	Up	Shoot	Anaerobiosis	[133]
		*ACS3*	Up	Root	Anaerobiosis	[133]
**15.**	*Petunia*	*ACS1*	Up	Leaves	Salt	[134]
		*ACO1*, *ACO3*	Up	Leaves	Salt/Drought	[134]
**16.**	*Pisum sativum*	*ACS4*, *ACO1*, *ACO2*	Up	Pre-pollinated ovaries	Heat	[135]
		*ACS4*	Up	Post-pollinated ovaries	Heat	[135]
		*ACS2*, *ACS4*, *ACO1*, *ACO3*	Up	Pedicel	Heat	[135]
		*ACO2*	Down	Pedicel	Heat	[135]
		*ACS2*	Up	Anthers	Heat	[135]
		*ACS2*, *ACO2*	Up	Stigma/style	Heat	[135]
		*ACS2*, *ACS4*, *ACO3*	Up	Petals	Heat	[135]
**17.**	*Saccharum officinale*	*ACO2*, *ACO5*	Up	Leaves	Drought	[136]
		*ACS*	No expression detected	Leaves	Drought	[136]
**18.**	*Solanum tuberosum*	*ACO1*	Up	Leaves	Flooding	[137]
		*ACO2*	Down	Leaves	Flooding	[137]
		*ACO1*	Up	Tubers	Heat/cold	[137]
		*ACO2*	Up	Tubers	Cold	[137]
**19.**	*Triticum aestivum*	*ACS1*, *ACS3*, *ACS7*, *ACS9*, *ACS10*, *ACS11*	Up	-*	Drought	[49]
		*ACS8*, *ACS6*	Down	-*	Drought	[49]
		*ACS7*, *ACS9*, *ACS10*	Up	-*	Salt	[49]
		*ACS1*, *ACS2*, *ACS3*, *ACS4*, *ACS5*, *ACS6*, *ACS8*, *ACS11*, *ACS12*	Up	-*	Cold	[49]
		*ACS10*	Down	-*	Cold	[49]
		*ACS4*, *ACS5*, *ACS6*	Up	-*	Heat	[49]
**20.**	*Zea mays*	*ACS1a*	Down	Leaves	Salt	[138]
		*ACO5b*	Up	Leaves	Salt	[138]
		*ACS2*, *ACS7*	Up	Root cortex	Hypoxic	[139]
		*ACO15/31*, *ACO20/35*	Up	Root cap	Hypoxic	[139]

GM-OE: genetically modified over-expressing; -*: not specified.

**Table 3:** Differential expression of various *ACS* and *ACO* isoforms in response to different nutritional stress.

S.No.	Plant Species	Gene Targeted	Up/Downregulated	Plant Organ	Nutritional Stress	References
**1.**	*Arabidopsis thaliana*	*ACS 4*, *ACS6*, *ACO1*, *ACO2*	Up	Root	Magnesium deficiency	[194]
		*ACS2*, *ACS4*, *ACS6*, *ACS7*	Up	Root	Phosphate deficiency	[187]
		*ACO*	Up	Root	Phosphate deficiency	[206]
		*ACS4*, *ACS6*, *ACS9*, *ACO1*, *ACO2*	Up	Root	Iron deficiency	[197]
		*ACS2*, *ACS6*, *ACS7*, *ACS8*, *ACS11*	Up	Leaves and root	Iron deficiency	[198]
		*ACS9*	Up	Roots	Iron deficiency	[198]
		*ACS2*, *ACS7*, *ACS8*	Up	Leaves	Magnesium deficiency	[192]
		*ACS11*	Up	Leaves and Roots	Magnesium deficiency	[192]
		*ACS2*, *ACS4-8*, *ACS11*, *ACO1*, *ACO2*	Up	Seedling	High nitrate	[182]
		*ACS6*	Up	Root	Nitrogen deficiency	[207]
		*ACO2*, *ACO4*	Up	Shoot	Nitrogen deficiency	[207]
		*ACS11*	Up	Root	Boron deficiency	[202]
		*ACS6*, *ACO2*, *ACO4*	Up	Leaves	Nitrogen deficiency	[208]
		*ACS2*, *ACS6*	Up	Root, leaves	Potassium deprivation	[209]
**2.**	*Brassica napus*	*ACS*, *ACO*	Up	Leaves	Sulfur deficiency	[200]
**3.**	*Citrullus lanatus*	*ACS9*, *ACS1*, *ACO1*	Down	Root	Potassium deprivation	[210]
		*ACO4*	Up	Roots	Potassium deprivation	[210]
**4.**	*Cucumis sativus*	*ACO*	Up	Leaves	Nitrogen deficiency	[180]
**5.**	*Medicago falcata*	*ACS*, *ACO*	Up	seedlings	Phosphorus deficient	[211]
**6.**	*Medicago sativa*	*ACO*	Up	Root	High nitrate	[212]
**7.**	*Medicago truncatula*	*ACS2*, *ACO*	Up	Root apices	Aluminium exposure	[213]
**8.**	*Lycopersicon esculentum*	*ACO1*	Up	Pedicel	Calcium treatment	[205]
**9.**	*Trifolium repens*	*ACS1*, *ACS2*, *ACS3*, *ACO1*, *ACO3*	Up	Root	Phosphate deficiency	[214]
